# Interactions between host sex and seasonal changes shape the gut microbial communities of wild blue sheep (*Pseudois nayaur*)

**DOI:** 10.3389/fmicb.2025.1553622

**Published:** 2025-05-16

**Authors:** Yaxin Dong, Zhirong Zhang, Zhaoling Zhu, Tianhua Hu, Junda Chen, Liwei Teng, Zhensheng Liu

**Affiliations:** ^1^College of Wildlife and Protected Areas, Northeast Forestry University, Harbin, China; ^2^College of Economics and Management, Jiamusi University, Jiamusi, China; ^3^Helan Mountain National Nature Reserve of Ningxia, Yinchuan, China; ^4^Key Laboratory of Conservation Biology, National Forestry and Grassland Administration, Harbin, China

**Keywords:** gut microbiome, seasonal changes, sex differences, wildlife conservation, 16S rRNA sequencing

## Abstract

The construction of gut microbial communities in wildlife is influenced by both environmental factors and host genetic background. However, the mechanisms through which these factors interact to shape microbial communities remain poorly understood. In this study, we systematically sampled fecal specimens from male and female wild blue sheep across different seasons in Helan Mountain Nature Reserve and analyzed them using 16S rRNA gene sequencing. The objective was to investigate seasonal changes and interactions between sex and season on the gut microbial communities of blue sheep. Our results revealed that Firmicutes and Bacteroidetes were the dominant phyla across all groups, and the ten most abundant genera remain stable across both sexes and seasons. Alpha diversity (Chao1, two-way ANOVA, *p* = 0.001) and Beta diversity (PCoA, Anosim, R = 0.5410, *p* = 0.001) analyses further confirmed that seasonal and sex-specific interactions significantly shape the microbial community structure. Notably, the gut microbiomes of male and female blue sheep exhibited distinct response patterns to seasonal changes. LEfSe analysis (LDA > 3) identified 20 microbial taxa with significant seasonal differences, some of which showed sex-specific seasonal variation. These findings highlight the critical role of host sex in modulating the adaptation of gut microbial communities to seasonal environmental stresses. This study provides new insights into the ecological and evolutionary mechanisms of wildlife gut microbiomes and offers a scientific basis for sex-based wildlife conservation strategies.

## 1 Introduction

Microbial colonization of the gut in wildlife forms microbial ecological communities that play crucial roles in host metabolism (Holmes et al., 2011; Visconti et al., 2019), immune function (Rizzetto et al., 2018), and physiological homeostasis (Lee et al., 2022). Typically, a dynamic equilibrium is maintained between the gut microbiome and the host (Fassarella et al., 2021; Lee et al., 2022). This balance is regulated by multiple factors, including the host’s genetic background, immune status, dietary composition, and other regulatory elements (Spor et al., 2011; Kurilshikov et al., 2017). However, environmental stressors, host stress, and other disturbances can disrupt this equilibrium, leading to changes in the structure of the microbial communities and the potential exacerbation of metabolic and immune dysfunction (Candela et al., 2012; Carding et al., 2015; Karl et al., 2018). Understanding the key factors influencing gut microbial community assembly and mechanisms underlying these interactions is essential for a deeper understanding of host-microbe relationships.

The construction and maintenance of gut microbial communities are regulated by both environmental factors and host genetics (Spor et al., 2011; Scepanovic et al., 2019). In wildlife, seasonal environmental changes, such as variations in food availability, temperature, precipitation, can significantly alter the diversity and structural composition of microbial communities (Nguyen et al., 2021; Stothart et al., 2024). For example, a recent study on Tibetan antelope (*Pantholops hodgsonii*) revealed significant seasonal shifts in both gut microbial composition and associated metabolic profiles between the cold and warm seasons (Zhao et al., 2023). These environmental changes not only directly affect microbial communities, but also indirectly influence them by modifying the physiological state and foraging behavior of the host (Badri et al., 2013; Trevelline and Kohl, 2022). Additionally, the genetic makeup of the host contributes to the composition of the gut microbiome (Khachatryan et al., 2008; Dai et al., 2022), shaping factors such as immune responses, metabolic processes, and gut microenvironment (Rooks and Garrett, 2016). Among genetic factors, sex-related differences in hormone levels (Org et al., 2016), immune responses (Johnson et al., 2020), and metabolic patterns (Valeri and Endres, 2021) are particularly influential in microbial community construction. The interaction between genetic factors and environmental influences thus determines the dynamic characteristics of gut microbial communities (Benson et al., 2010; Spor et al., 2011).

While the interactions between environmental factors and microbial communities have been extensively studied, the role of host genetics, particularly sex-specific differences, remains largely unclear (Maritan et al., 2024). In wildlife, males and females often exhibit different ecological responses to seasonal changes, such as variations in foraging behavior, habitat selection, and social interactions (Bronson, 1985; Stinson, 1985; Munday et al., 2006; La Reau and Suen, 2018; Stoffel et al., 2020). These differences can have significant implications for gut microbiome composition, as sex-specific environmental adaptations might shape microbial colonization patterns in unique ways (DeCandia et al., 2024). Moreover, the effects of sex differences may be more pronounced in resource-limited environments, where ecological pressures are higher and may amplify the effects of sex on microbial community dynamics (Maurice et al., 2015; Hernández et al., 2024). Therefore, understanding how seasonal changes and sex interact to shape microbial communities in wildlife is essential for uncovering the evolutionary processes that govern host–microbe interactions.

The gut microbiomes of wildlife species are highly susceptible to seasonal and sex-specific environmental pressure. Here we explore the effects of seasonal variation and sex interactions on gut microbial communities of wild blue sheep (*Pseudois nayaur*) populations in Helan Mountain Reserve, using 16S rRNA gene sequencing to analyze microbial composition. Specifically, this study aims to: (I) examine whether compositional changes in the gut microbial community differ between male and female blue sheep across seasonal transitions; (II) assess whether seasonal shifts lead to structural differences in the gut microbial communities of males and females; and (III) identify sex-specific marker taxa. Through these objectives, we aim to elucidate how environmental factors and host genetic traits, particular sex, interact to shape wildlife gut microbiomes, providing insights into microbial ecology and informing conservation strategies.

## 2 Materials and methods

### 2.1 Study site and sample collection

This study was conducted in the Helan Mountain National Nature Reserve (E 105°49′ ~ 106°42′, N 38°21′ ~ 39°22′) in northwest China, a transitional zone between the northern temperate grasslands and temperate deserts. The reserve encompasses a southwest-northeast mountain range with a slightly curved strike, spanning 20–40 km in width from east to west and approximately 250 km in length from north to south. The region experiences high summer temperatures exceeding 40°C during the day and cold winters, with minimum temperatures reaching approximately −20°C. Annual precipitation is sparse, primarily occurring in summer. These climatic conditions create a unique habitat that supports a diverse array of flora and fauna, with blue sheep as the dominant species (Cui et al., 2024). The ecological roles and population dynamics of blue sheep are crucial indicators of the region’s ecological health and form the focus of this study. Sample collection was carried out in July (Summer) and December (Winter) of 2017 to examine seasonal variations in the gut microbial communities of blue sheep. Observers used double-pass binoculars to monitor blue sheep in their natural habitat from a distance, ensuring minimal disturbance. Fecal samples were collected immediately after defecation and only after the animals had vacated the site, ensuring samples freshness and representativeness. The same habitat areas were selected for both seasons to enable a comparative analysis. In total, 361 fresh fecal samples were collected during the two sampling periods.

### 2.2 Sex identification and sample selection

In this study, DNA was extracted from blue sheep fecal samples using the QIAamp Fast DNA Stool Mini Kit (QIAGEN, Hilden, Germany) and amplified via multiplex PCR with sex-determining primers SRY12 and BMCl009 (Huber et al., 2002). The PCR protocol included an initial denaturation at 94°C for 2 min, followed by 35 cycles of denaturation at 98°C for 10 s, annealing at 54°C to 60°C for 30 s, and extension at 68°C for 30 s. The final extension was performed at 68°C for 7 min. Sex determination was based on the presence of specific amplification band: male samples exhibited double bands (180 bp and 300 bp) in at least 2 of 3 parallel amplifications, while female samples showed a single band (300 bp) under same conditions. Following sex determination, 81 samples were randomly selected from 361 collected samples for subsequent analysis, including 19 summer females (SF), 22 winter females (WF), 21 summer males (SM) and 19 winter males (WM). This stratified sampling ensured balanced representation across seasons and sexes for subsequent analyses.

### 2.3 DNA extraction and 16S rRNA sequencing

The genomic DNA was extracted from the 81 selected blue sheep fecal samples using CTAB method (Zhu et al., 2020). The quality of the extracted DNA and PCR products were assessed via agarose gel electrophoresis (1% for DNA, 2% for PCR products). The quality-qualified DNA was stored at −20°C for future analysis. The V4 region of the 16S rRNA gene was amplified using universal bacterial primers 515F (5’-GTGCCAGCMGCCGCGGGTAA-3′) and 806R (5’-GGACTACHVGGGTWTCTAAT-3′) (Yan et al., 2024). PCR amplifications were performed using the Phusion® High-Fidelity PCR Master Mix with GC Buffer (New England Biolabs) and High-fidelity DNA polymerase. Each reaction contained 10 μL of High-Fidelity Enzyme, approximately 150 ng, and 0.5 μM of each two primers. The reaction program was 98°C pre denaturation for 30 s (1×); 98°C denaturation for 5–10 s, 55°C annealing for 10–30 s, 72°C extension for 2 min (35×); and final extension at 72°C for 20 min. The library was constructed using the Ion Plus Fragment Library Kit (Thermo Fisher Scientific, Waltham, MA, USA). The libraries were quantified using Qubit@ 2.0 (Thermo Fisher Scientific) and assessed for quality. Sequencing was performed on the Ion S5TM XL platform, generating 400 bp single-ended reads for downstream analysis (Zhang et al., 2023).

### 2.4 Bioinformatics analysis

The sequencing reads were processed using a standardized bioinformatics pipeline. Quality filtering was conducted using Cutadapt (v1.9.1), during which sequencing reads were demultiplexed based on Barcode, and Barcode and primer sequences were removed to generate raw data (Martin, 2011). After preliminary quality control, the raw data were compared against a species annotation database, and chimeric sequences were removed to obtain clean reads. Noise reduction and amplicon sequence variation (ASV) inference were performed in QIIME2 using the DADA2 module (Fung et al., 2021). The obtained ASVs were compared with the Silva 138.1 database to annotate taxonomic information (Lee, 2024). Species identification was conducted using classify-sklearn algorithm in QIIME2, which employs a trained Naive Bayes classifier. This process yielded detailed information on species abundance and distribution based on the annotated ASVs.

### 2.5 Statistical analysis

Alpha diversity indices, including Chao1, Shannon and Simpson indices, were calculated using the ‘phyloseq’ and ‘vegan’ packages to assess community richness and evenness at the ASV level. Prior to calculation, ASV tables were rarefied to the minimum sequencing depth across samples to account for uneven sampling effort. The effects of season (summer and winter), sex (male and female) and their interactions on diversity indices were analyzed using two-way ANOVA. Community structure differences at the ASV level were evaluated using principal coordinate analysis (PCoA) based on Bray-Curtis distances, with statistical significance assessed via the analysis of similarities (ANOSIM) test using the ‘vegan’ package. To quantify the relative contributions of season and sex to community variation, redundancy analysis (RDA) was employed at the ASV level using ‘vegan’ package. Hierarchical cluster analysis (HCA) was used to explored phylogenetic relationships among treatment groups. Differential microbial analysis was performed using LEfSe (Linear discriminant analysis Effect Size) to identify microbial taxa with significant seasonal patterns (Segata et al., 2011). The analysis was conducted through the online platform of Majorbio Cloud Platform following default settings (LDA score > 3). The Kruskal-Wallis test was applied to examine whether these taxa the taxa identified by LEfSe exhibited seasonal differences across sexes, and genus-level differences was verified using the Wilcoxon rank-sum test. To further assess the sex-specific microbial response to seasonal changes, support vector machine (SVM) was conducted at the ASV level, withe model accuracy s verified through cross-validation (Topçuoğlu et al., 2020). All analyses were completed using the online platform of Majorbio Cloud Platform and R software (v4.3.2) packages, including vegan (v2.6.4) for diversity analysis, phyloseq (v1.44.0) for data normalization, DESeq2 for differential analysis, and ggplot2 (v3.4.2) for data visualization. The significance level was set at *p <* 0.05.

## 3 Results

### 3.1 Seasonal and sex-specific differences in gut microbial composition

**Figure 1:**
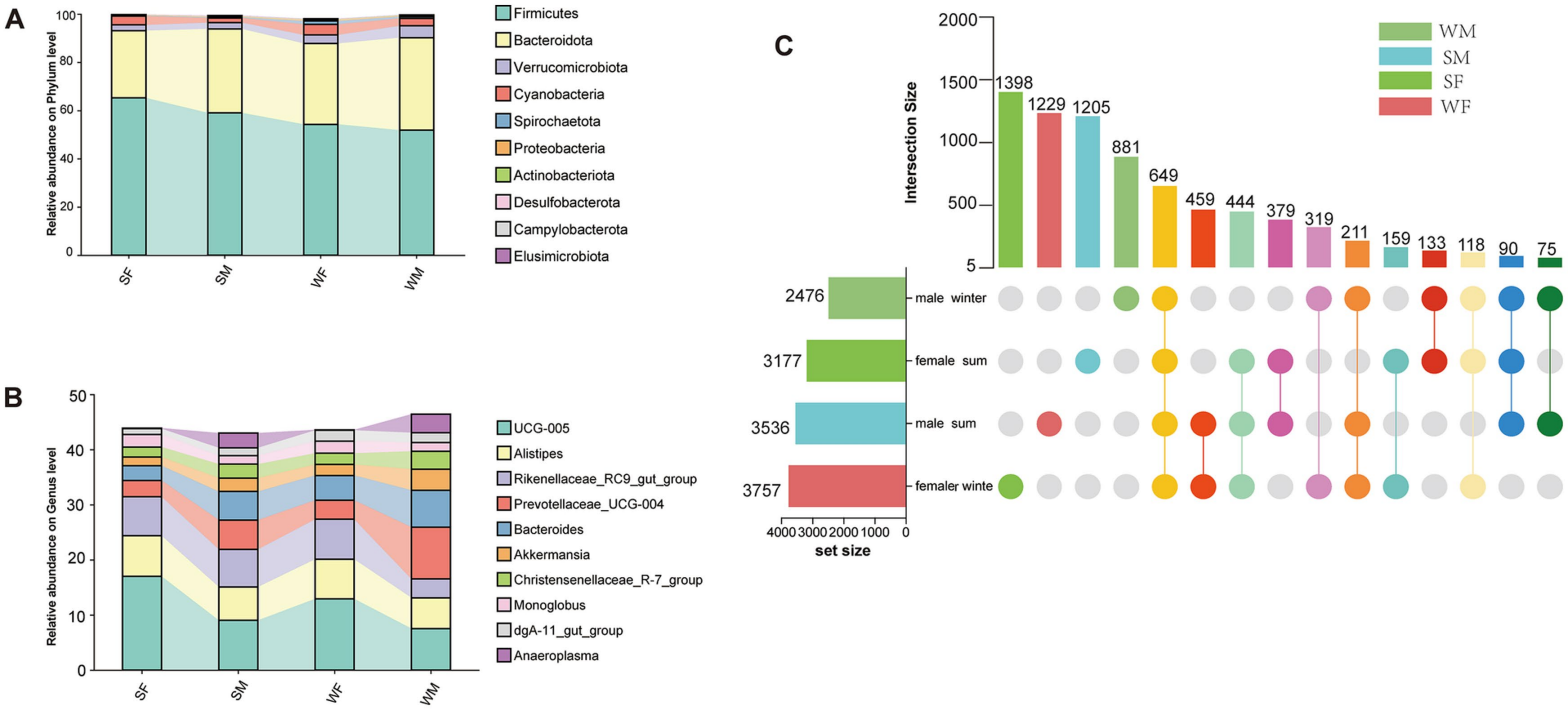
Sex-specific seasonal dynamics of gut microbiota composition and core microbiome in wild blue sheep. **(A)** Phylum-level taxonomic composition of the gut microbiota across different seasons and sexes, showing the dominance of Firmicutes and Bacteroidetes. **(B)** Relative abundance of the top 10 most abundant bacterial genera, revealing distinct seasonal and sex-specific patterns in microbial community structure. **(C)** UpSet plot demonstrating the shared and unique bacterial ASVs among different sex-season combinations. The horizontal bars represent the total number of ASVs in each group, while the vertical bars indicate the size of intersecting sets. The connected dots below show the specific combinations of shared ASVs.

A total of 7,769 ASVs were identified from 81 samples based on 16S rRNA gene sequencing, spanning 18 phyla, 112 families, and 226 genera. The dominant phyla Firmicutes, Bacteroidetes, and Proteobacteria were consistently abundant across seasons and sexes (Figure 1A). The top ten genera by abundance remained stable across all groups and included *UCG-005*, *Alistipes, Rikenellaceae RC9 gut group*, *Prevotellaceae UCG-004*, *Bacteroides*, *Akkermansia*, *Christensenellaceae R-7 group*, *Monoglobus*, *dgA-11 gut group*, and *Anaeroplasma* (Figure 1B). Upset plot analysis revealed seasonal and sex-specific differences in the number of ASVs that indicated the total number of ASVs for all samples in each sex-season combination. In female, a greater number of ASVs was detected in winter (3,757 ASVs) compared to summer (3,177 ASVs), with unique ASVs also showing significant seasonal variation (1,222 in winter vs. 1,398 in summer) (Figure 1C). Conversely, male exhibited the opposite trend, with higher ASVs counts in summer (3,536 ASVs) than in winter (2,476 ASVs), and a marked seasonal difference in unique ASVs (1,205 in summer vs. 881 in winter) (Figure 1C). These patterns suggest a seasonal shift in gut microbial diversity, with females showing enhanced microbial richness in winter, while males exhibit greater diversity in summer. Across all groups, 649 ASVs were identified that were shared across all four sex-season combinations (SM, WM, SF, and WF), indicating a common microbial subset potentially contributing to gut ecosystem stability.

### 3.2 Sex differences in seasonal variation of gut microbial *α*-diversity in blue sheep

**Figure 2:**
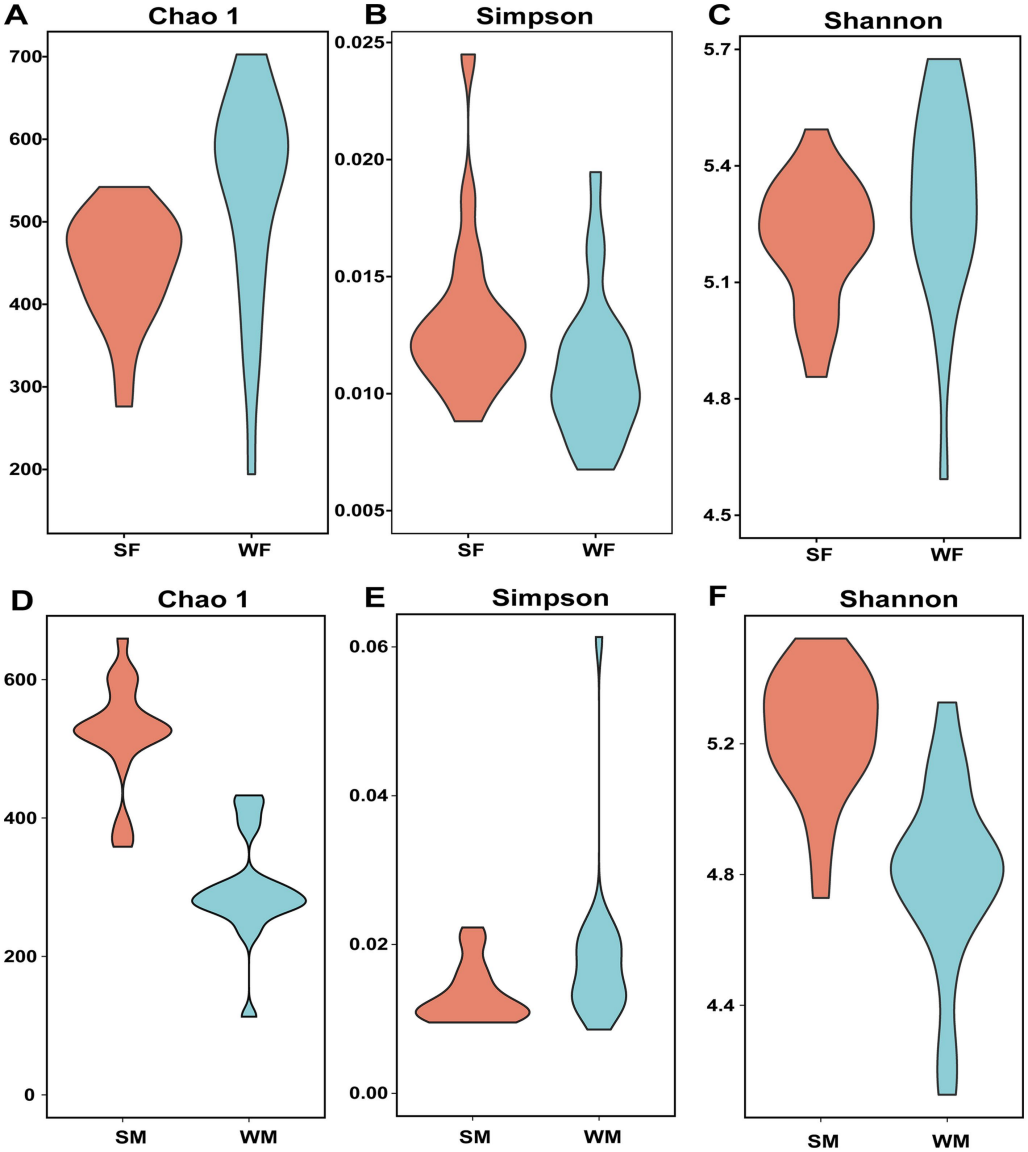
Sex-specific seasonal shifts in gut microbiota diversity reveal distinct ecological strategies between male and female blue sheep. Alpha diversity reveals contrasting seasonal patterns between sexes in the gut microbiota of wild blue sheep. Female seasonal changes in microbial diversity: **(A)** Chao1, **(B)** Simpson, and **(C)** Shannon indices between summer (SF) and winter (WF) samples. Male seasonal changes in the same diversity indices between summer (SM) and winter (WM) samples: **(D)** Chao1, **(E)** Simpson, and **(F)** Shannon.

To assess microbial richness and evenness at the ASV level, we calculated ASV counts along with the Chao1, Shannon, and Simpson indices. Due to the strong mathematical correlation between ASV counts and the Chao1 index, we primarily reported the Chao1 index when evaluating species richness, as it offers a more robust estimate by accounting for rare and low-abundance taxa. A two-way ANOVA of the Chao1 index revealed that the interaction between season and sex was the main driver of microbial diversity differences across groups (*p* = 0.001) (Figure 2; Table 1). Specifically, female blue sheep showed a significantly higher Chao1 index in winter compared to summer (*p <* 0.05), while males displayed a significantly higher Chao1 index in summer than in winter (*p <* 0.001) (Figures 2A,D; Supplementary Table S1). The Shannon index analysis demonstrated a significant interaction effect between season and sex on gut microbial diversity (*p =* 0.001) (Figure 2; Table 1). The Shannon index was significantly higher in male blue sheep during the summer than winter (*p* = 0.001), whereas females exhibited no significant seasonal variation (*p* > 0.05) (Figures 2C,F; Supplementary Table S1). Simpson Index analyses revealed marked seasonal and gender interactions, which influenced the balance of the microbial community (*p* < 0.05) (Figure 2; Table 1). Males showed a higher Simpson index in winter, suggesting a greater dominance of specific microbial populations in their gut communities compared to other groups (Figures 2B,E; Supplementary Table S1).

**Table 1:** Two-way ANOVA to detect the effect of season and sex interaction on the alpha diversity of gut microorganisms.

*α* Diversity	Term	df	Sumsq	Meansq	Statistic	*p*-value
Chao1	Season	1.00	76480.46	76480.46	9.15	0.003
	Sex	1.00	131713.13	131713.13	15.75	0.001
	Season:sex	1.00	499806.32	499806.32	59.78	0.001
	Residuals	77.00	643778.09	8360.75		
Shannon	Season	1.00	0.44	0.44	8.10	0.005
	Sex	1.00	1.24	1.24	22.76	0.001
	Season:sex	1.00	1.42	1.42	26.08	0.001
	Residuals	77.00	4.19	0.05		
Simpson	Season	1.00	0.00	0.00	0.74	0.392
	Sex	1.00	0.00	0.00	7.55	0.007
	Season:sex	1.00	0.00	0.00	6.70	0.011
	Residuals	77.00	0.00	0.00		

### 3.3 Seasonal and sex driven microbial community differentiation

**Figure 3:**
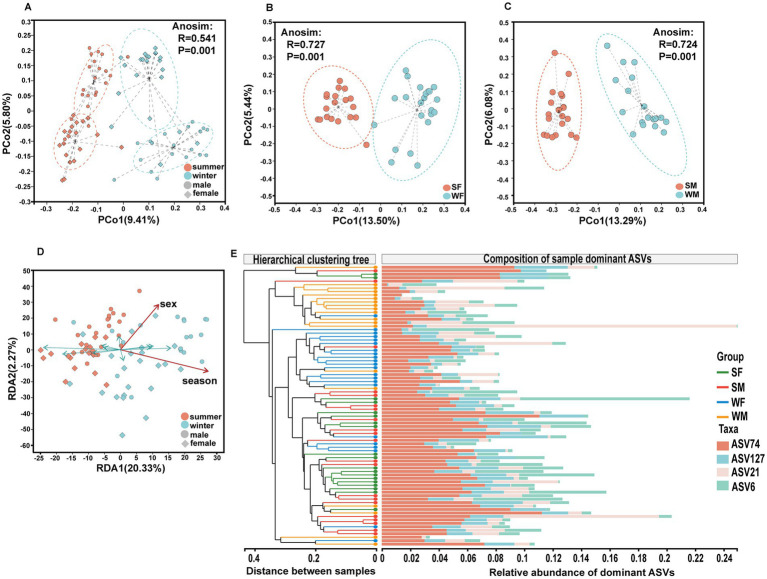
Multidimensional analysis reveals distinct seasonal and sex specific structuring of gut microbiota communities in wild blue sheep.**(A)** Principal Coordinates Analysis (PCoA) based on Bray-Curtis distances demonstrates significant clustering of gut microbiota communities by both season and sex; Sex-specific seasonal differences in community structure shown separately for females **(B)** and males **(C)**, revealing equally strong but distinct patterns of seasonal community shifts between sexes. **(D)** Redundancy Analysis (RDA) illustrating the relative contributions of sex and seasonal factors in shaping microbial community composition, with arrows indicating the direction and magnitude of environmental variables. **(E)** Hierarchical clustering analysis combined with relative abundance heatmap of core ASVs across different sex-season groups.

Principal coordinate analysis (PCoA) based on the Bray-Curtis distance at the ASV level revealed significant differentiation between subgroups (Anosim, *R =* 0.5410, *p =* 0.001), with clear separation between winter and summer samples (Figure 3A). In single sex subgroups, both females (Anosim, *R =* 0.7266, *p =* 0.001) and males (Anosim, *R =* 0.7242, *p =* 0.001) showed significant seasonal community differentiation, with greater similarity within each sex. For females, PCo1 and PCo2 explained 13.50 and 5.44% of the variance, respectively, while for males, PCo1 and PCo2 accounted for 13.29 and 6.08% of the variance (Figures 3B,C). Redundancy analysis (RDA) further quantified the effects of sex and season on community structure, with RDA1 (20.33%) and RDA2 (2.27%) together explained 22.60% of the total variation (Figure 3D). It is noteworthy that seasonal factors were primarily reflected in RDA1 axis, while sex differences were more pronounced along RDA2. Hierarchical cluster analysis revealed that summer samples (SM and SF) showing higher community similarity, whereas winter samples (WM and WF) showed greater within-group variability (Figure 3E).

### 3.4 Sex-dependent seasonal changes in gut microbial taxa and ASVs

**Figure 4:**
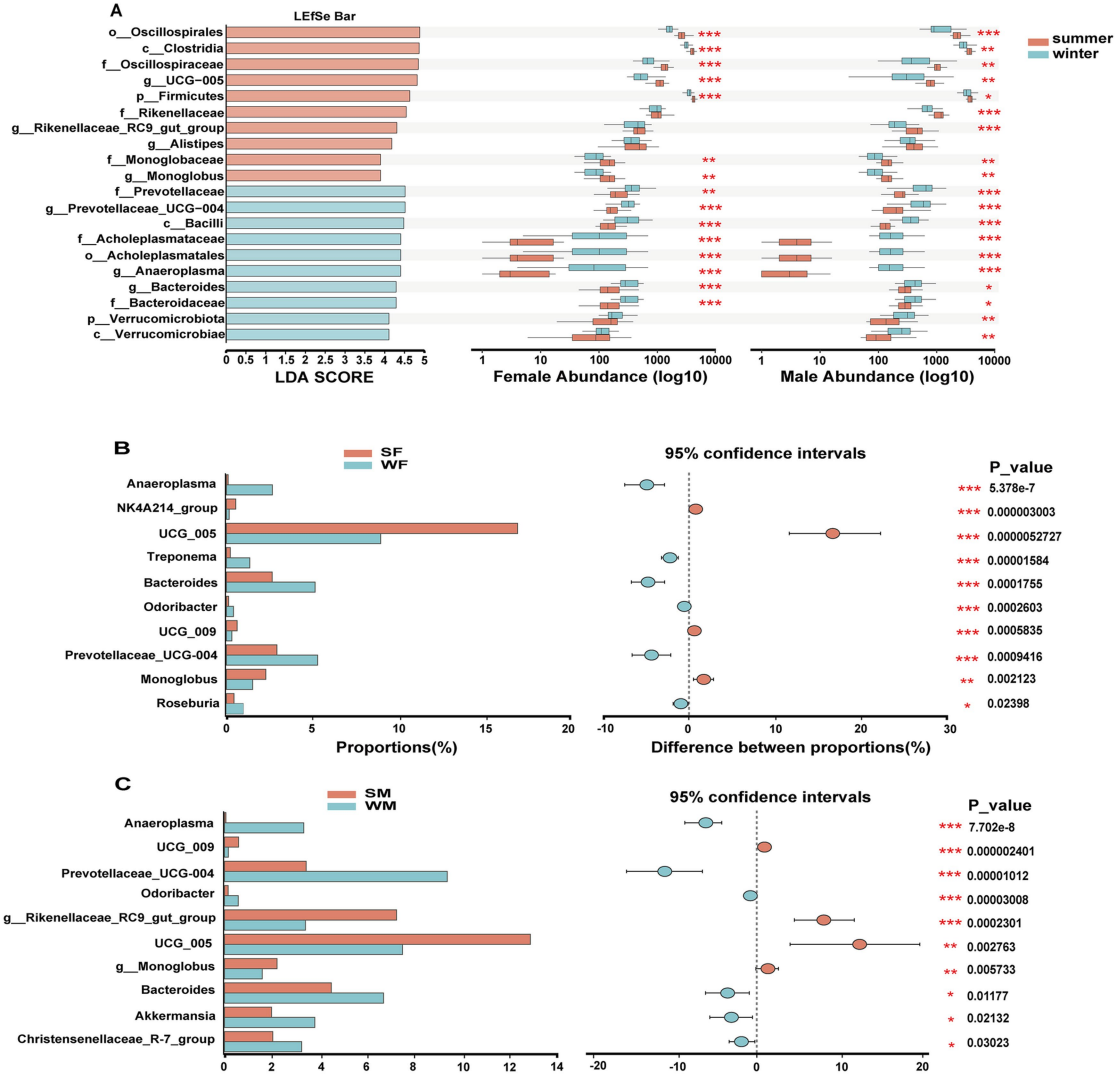
Sex specific seasonal restructuring of gut microbiota reveals distinct taxonomic signatures and adaptation strategies in wild blue sheep. **(A)** LEfSe analysis identifying season-discriminative taxa across taxonomic levels (left panel), with Kruskal-Wallis test results showing seasonal abundance variations separately for females and males (right panels). Differential abundance analysis of key bacterial genera between seasons, shown separately for females **(B)** and males **(C)**. Left panels show relative abundances (%), while right panels display the magnitude and direction of seasonal shifts with 95% confidence intervals. Statistical significance is indicated by asterisks (*p < 0.05, **p < 0.01, ***p < 0.001).

**Figure 5:**
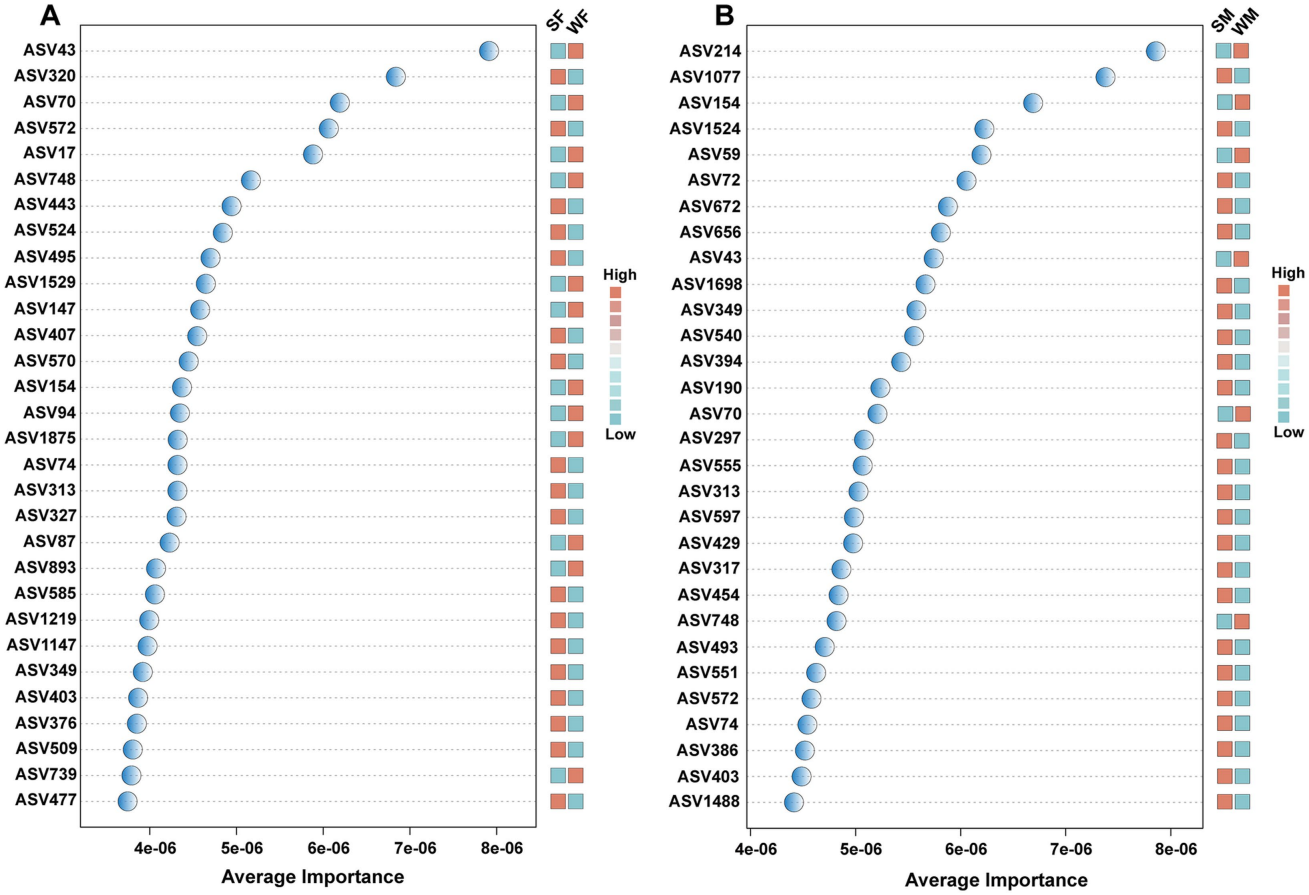
Support Vector Machine (SVM) analysis identifying discriminative ASVs that drive seasonal patterns in female **(A)** and male **(B)** blue sheep. Average importance scores (x-axis) represent the contribution of each ASV to the SVM classification model’s performance in distinguishing seasonal patterns. Adjacent heatmaps display the relative abundance patterns of each ASV across seasonal groups.

LEfSe analysis identified 20 microbial taxa with significant seasonal differences, including taxa from phylum to genus levels, of which 9 taxa were enriched in the summer and 11 in the winter. The Kruskal-Wallis test further revealed that these taxa exhibited different seasonal response patterns between males and females, with 4 taxa showing distinct seasonal variation between the sexes (Figure 4A). Specifically, in males, *Verrucomicrobiota, Verrucomicrobiae, Rikenellaceae, Rikenellaceae RC9 gut group* showed significant seasonal differences that were not observed in females (Figure 4A). The Wilcoxon rank sum test revealed significant sex-specific seasonal variation in differential genera. In females, the genera *NK4A214 group*, *Treponema*, and *Monoglobus* were seasonally differentiated, while in males, the genera *Rikenellaceae RC9 gut group, Akkermansia*, and *Christensenellaceae R-7 group* genera were identified as male-specific seasonal markers (Figures 4B,C). Support vector machine (SVM) analysis based on ASV-level abundance profiles confirmed these sex-specific seasonal pattern. Most of the ASVs identified by SVM with the highest discriminatory power belonged to either *Clostridia* or *Oscillospirales* highlighted in LEfSe, and at the genus level, ASV320, ASV407, ASV74, ASV313, and ASV327 were all annotated as *UCG-005*, which is also a genus highlighted in LEfSe. Among the top 30 most discriminative ASVs, only eight ASVs were common to both females and males (ASV43, ASV70, ASV74, ASV154, ASV313, ASV349, ASV403, and ASV572). In addition, 12 ASVs exhibited higher abundance in winter in females (Figure 5A), whereas only six ASVs showed this pattern in males (Figure 5B).

## 4 Discussion

In the context of global climate change, increasing attention has been given to the role of seasonality in shaping the gut microbial communities of wildlife (Guan et al., 2023; Williams et al., 2023). While many studies have primarily examined seasonal differences in gut microbial community composition, the interaction between seasonal environmental factors and host genetic traits remains underexplored (Spor et al., 2011; Bolnick et al., 2014). In this study, we used wild blue sheep of both sexes as model organisms to investigate how season and sex jointly influence the construction of wildlife gut microbial communities. Our findings revealed significant seasonal differences in the gut microbial community composition of wild blue sheep, observed consistently across both male and female individuals. Notably, while both sexes exhibited significant seasonal variation in community structure and characteristic genera, the patterns of these variations differed between males and females. The sex-specific patterns in gut microbial community structure, despite exposure to identical seasonal environmental pressures, reveal fundamentally distinct adaptive strategies between male and female blue sheep. These findings indicate that seasonal changes in gut microbial abundance observed in male blue sheep cannot be directly extrapolated to females.

In response to seasonal changes, we observed a high degree of similarity in the composition of dominant phyla and genera between female and male wild blue sheep. This finding aligns with previous studies suggesting that the core microbiota of wildlife gut communities generally exhibit significant stability under natural environmental conditions, provided there are no severe disturbances such as extreme weather events or diseases (Jiang et al., 2021; Ma et al., 2024; Nanetti et al., 2024). In our study, Firmicutes and Bacteroidetes consistently represented the most dominant phyla, a pattern commonly observed in other ungulate species (Jiang et al., 2021; Gao et al., 2024). The stability of this core microbiota is likely influenced by host genetic factors, which play a crucial role in maintaining key microbial populations. As major components of the core microbiota, Firmicutes and Bacteroidetes contribute critically to food digestion, energy metabolism, and the maintenance of the intestinal immune barrier—functions essential for the survival of wild blue sheep (Guan et al., 2017). Under relatively stable environmental conditions, preserving the stability of the core microbiota may represent a vital strategy for ensuring the host’s physiological homeostasis. While the core microbiota displayed a high degree of conservatism, we identified specific microbial taxa with sex-dependent seasonal response patterns. LEfSe analyses revealed significant seasonal patterns in gut microbial dynamics. Notably, higher taxonomic ranks such as *Verrucomicrobiota* and *Verrucomicrobiae* showed male-exclusive seasonal variations, suggesting fundamental differences in microbial community restructuring between sexes. At finer taxonomic resolution, the sex-specific seasonal response patterns became more pronounced, with distinct sets of indicator genera characterizing male and female responses. Different seasonal signatures were observed between sexes, with *NK4A214 group*, *Treponema*, and *Monoglobus* showing female-specific seasonal variations, while *Rikenellaceae RC9 gut group*, *Akkermansia*, and *Christensenellaceae R-7 group* demonstrated male-specific seasonal shifts. These sex-specific marker genera likely correspond to physiological requirements unique to each sex. For example, *Akkermansia*, known for its roles in energy metabolism and intestinal barrier maintenance (Mo et al., 2024), showed seasonal variation in males, possibly reflecting the heightened energy demands during the mating season. Similarly, the seasonal variation of *Treponema* in females may be associated with the regulation of nutritional requirements during reproductive cycles (Dos Santos et al., 2024).

The gut microbial community structure of wild blue sheep showed significant changes with season, a trend consistent with the seasonal dynamics of the gut microbiome observed in other wild animals (Ren et al., 2017; Jiang et al., 2021). In addition to seasonal dynamics, blue sheep of different sexes exhibited distinct microbial community restructuring patterns, suggesting sex-specific responses to seasonal changes. The contrasting seasonal diversity patterns between males and females, coupled with distinct community compositions, suggest fundamentally different adaptive strategies between sexes. Sex-specific seasonal responses may reflect differential strategies adopted by male and female individuals in response to different ecological stresses (Aryal et al., 2014; Meng et al., 2023). For females, they experience significant physiological changes around the breeding season, including hormonal fluctuations and increased energy demands (Cizauskas et al., 2015; Sontakke, 2018). Given that blue sheep typically give birth in the warm season (Zhen-sheng et al., 2005), females exhibit higher microbial diversity during the winter, possibly in response to the demands of pregnancy and lactation. In contrast, male ungulates exhibit more frequent territorial patrolling and competitive behavior during the mating season, which requires the support of a more efficient energy conversion system (Owen-Smith, 1977; Forsyth et al., 2005; Bowyer et al., 2020). Male blue sheep show higher diversity in the summer, which may be related to their need to maintain higher fitness levels during the mating season.

The assembly of wildlife gut microbial communities is regulated by both environmental factors (e.g., climate, food resources) and host genetic characteristics (Spor et al., 2011; Bolnick et al., 2014). Understanding these regulatory mechanisms has become increasingly critical for wildlife conservation, particularly in the context of accelerating global climate change. While extensive research has examined the interactions between environmental factors and host genetic traits, most studies have been confined to laboratory settings or human subjects (Benson et al., 2010; Bolnick et al., 2014), limiting their applicability to complex natural environments, where investigating such interactions presents significant methodological challenges. Consequently, wildlife research has predominantly focused on examining interactions among various environmental factors, such as elevational and seasonal variations (Tang et al., 2023), with less emphasis on environment-host genetic interactions. Among the multiple influential factors, seasonal patterns and sex-specific differences emerge as both quantifiable and significant determinants of microbial community composition. The present study therefore selected season and sex as focal variables, representing external environmental pressure and sex-based physiological characteristics respectively, to investigate their interactive effects on gut microbial community structure in wild blue sheep. The observed sex-specific responses to seasonal variations may serve as seasonal variations may serve as a crucial mechanism for population stability maintenance. Climate change is significantly altering seasonal patterns in highland regions (Beniston, 2003; Buytaert et al., 2011). Such alterations may disrupt the evolutionarily established sex-specific response mechanisms. Thus, incorporating sex-specific physiological and ecological requirements into conservation strategies is necessary to effectively mitigate the potential impacts of climate change on sex-specific seasonal responses. Although this study elucidated how environmental factors and host genetic traits jointly shape the gut microbial communities of wildlife, the absence of systematic environmental data—such as detailed records of plant resource availability or temperature fluctuations—may have limited our ability to fully interpret microbial ecological responses. Furthermore, while 16S rRNA gene sequencing effectively captures taxonomic shifts within microbial communities, its capacity to predict functional traits remains limited. To gain a more comprehensive understanding of microbial functional dynamics, future studies should integrate metagenomic and transcriptomic approaches.

## 5 Conclusion

This study investigates the mechanisms through which host genetic factors and environmental conditions interact to shape animal gut microbial communities in the wild. The research focused on seasonal differences in the gut microbial communities of male and female wild blue sheep, aiming to elucidate the interaction between season and sex in shaping these communities. The dominant phyla and genera of bacteria remained consistent across seasons for both sexes, but the overall microbial community structure exhibited distinct seasonal response patterns in male and female blue sheep. Specifically, female blue sheep showed higher microbial diversity in winter, whereas male blue sheep reached peak diversity in summer. Additionally, the study identified microbial taxa with significant seasonal differences, some of which exhibited sex-specific seasonal responses, either in females or males. These findings highlight the significant role of host sex in regulating the adaptation of gut microbial communities to seasonal environmental stresses. This underscores the importance of considering sex differences in addressing challenges posed by climate change and ecological conservation. Overall, this study highlights the dynamic shifts in the gut microbial communities of wild blue sheep driven by the interaction between sex and season, underscoring the pivotal role of host sex in modulating microbial ecological adaptation. Future research should integrate multi-omics approaches to elucidate the functional consequences of these microbial shifts on host metabolism, physiology, and environmental adaptation. In addition, expanding the geographic scope and including more species will be essential for evaluating the generalizability of these ecological patterns, ultimately providing a forward-looking theoretical framework for sex-specific strategies in wildlife conservation.

## Data Availability

The datasets presented in this study can be found in online repositories. The names of the repository/repositories and accession number(s) can be found at: https://www.ncbi.nlm.nih.gov/, NCBI/SRA accession/PRJNA602195.
