# Supplementary material for: Interactions between host sex and seasonal changes shape the gut microbial communities of wild blue sheep (Pseudois nayaur)
Source: Front Microbiol. 2025 May 16;16:1553622. doi: 10.3389/fmicb.2025.1553622 (PMC12122755; doi:10.3389/fmicb.2025.1553622)
Supplement: Supplementary file 1 [file Table_1.docx]

Supplementary Material

Seasonal variation and host sex combine to shape the gut microbial community of wild blue sheep (*Pseudois nayaur*)

Yaxin Dong^1^, Zhirong Zhang^1^, Zhaoling Zhu^2^, Tianhua Hu^3^, Junda Chen^1^, Liwei Teng^1,4*^ and Zhensheng Liu^1,4*^

^1^College of Wildlife and Protected Areas, Northeast Forestry University, Harbin, China

^2^College of Economics and Management, Jiamusi University, Jiamusi, China

^3^Helan Mountain National Nature Reserve of Ningxia, Yinchuan, China

^4^Key Laboratory of Conservation Biology, National Forestry and Grassland Administration, Harbin, China

*** Correspondence:**Liwei Teng

tenglw1975@163.com

Zhensheng Liu

zhenshengliu@163.com

# Supplementary Figures and Tables

## Supplementary Figures


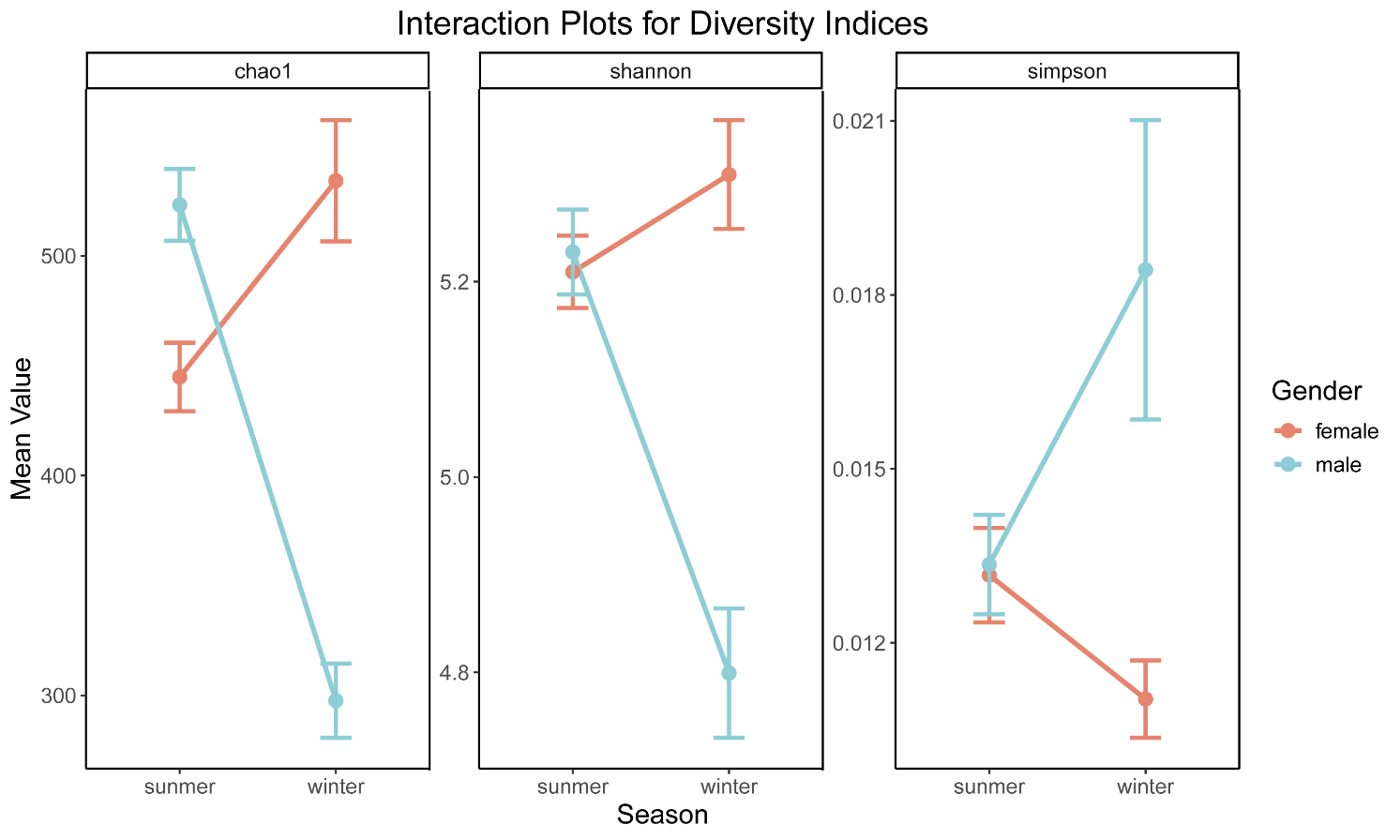


**Supplementary Figure 1.** Figure 1: Sex-specific seasonal dynamics in gut microbiota diversity of wild blue sheep reveals contrasting adaptive strategies. Interaction plots demonstrating sex-dependent seasonal variations in gut microbial diversity indices (Chao1, Shannon, and Simpson) of wild blue sheep (*Pseudois nayaur*).

**
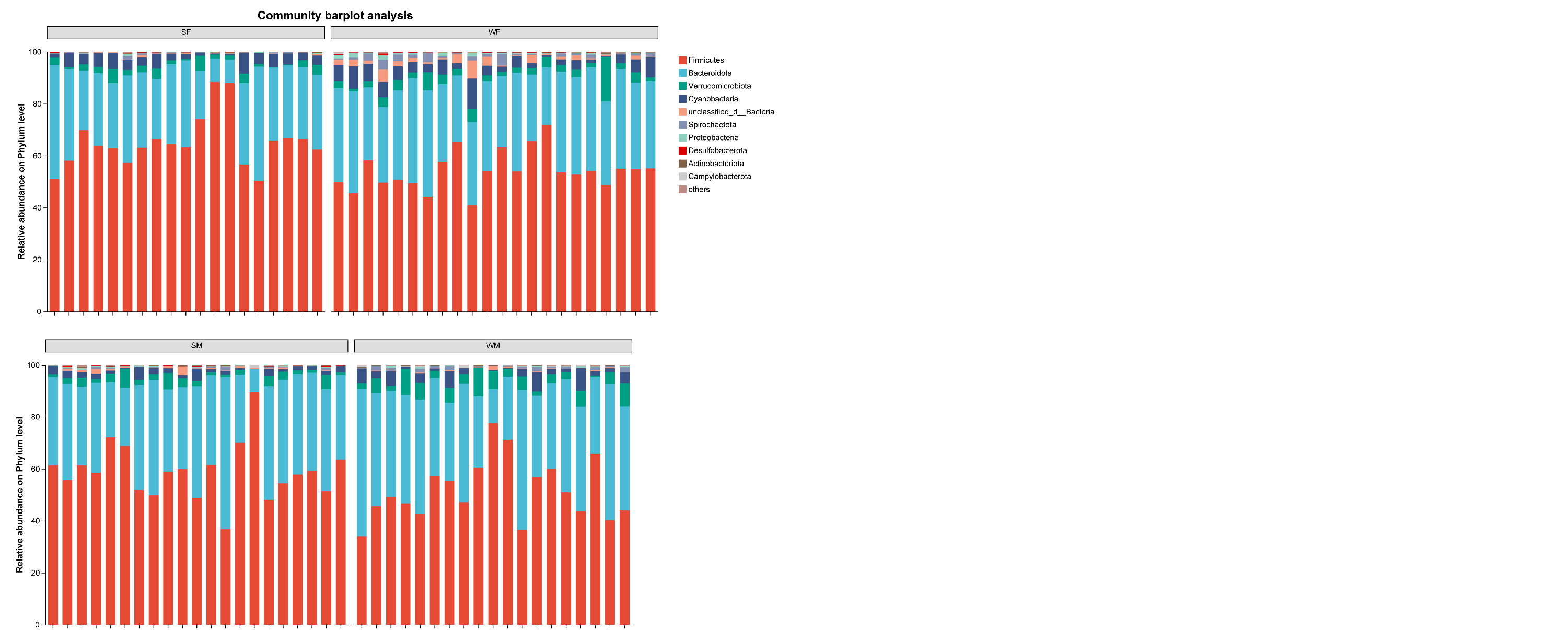
**

**Supplementary Figure 2.** Phylum-level taxonomic composition of the gut microbiota of all samples from different seasons and genders.

**
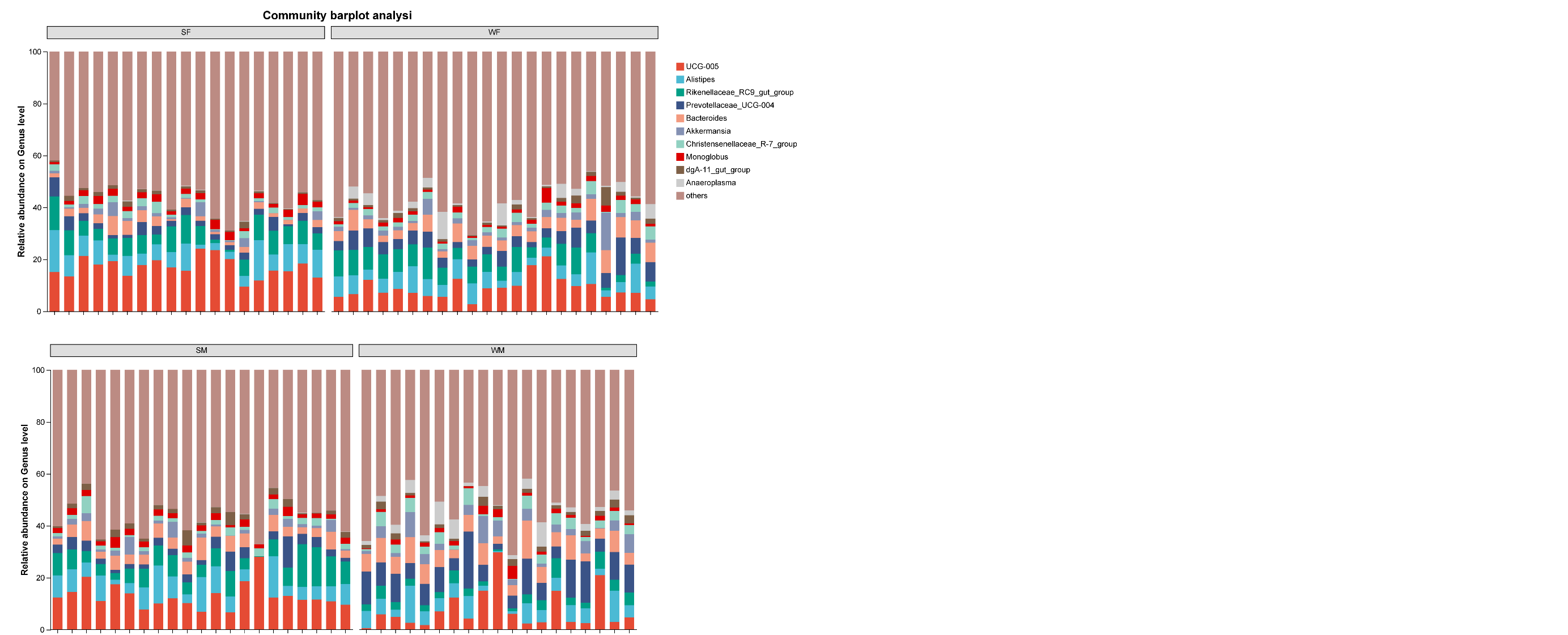
**

**Supplementary Figure 3.** Geuns-level taxonomic composition of the gut microbiota of all samples from different seasons and genders.

## Supplementary Tables

**Supplementary Table 1.** Intergroup pairwise results of Chao1、Shannon、Simpson indices of intestinal microbial communities in wild blue sheep of different sexes and seasons, with significance markers for differences at *p < 0.05*

| **α diversity** | **contrast** | **estimate** | **SE** | **df** | **t.ratio** | **p.value** |
| --- | --- | --- | --- | --- | --- | --- |
| Chao1 | summer female - winter female | -89.27 | 28.64 | 77.00 | -3.12 | 0.013 |
|  | summer female - summer male | -78.33 | 28.95 | 77.00 | -2.71 | 0.041 |
|  | summer female - winter male | 147.25 | 29.67 | 77.00 | 4.96 | 0.001 |
|  | winter female - summer male | 10.94 | 27.90 | 77.00 | 0.39 | 0.979 |
|  | winter female - winter male | 236.52 | 28.64 | 77.00 | 8.26 | 0.001 |
|  | summer male - winter male | 225.58 | 28.95 | 77.00 | 7.79 | 0.001 |
| Shannon | summer female - winter female | -0.10 | 0.07 | 77.00 | -1.36 | 0.526 |
|  | summer female - summer male | -0.02 | 0.07 | 77.00 | -0.28 | 0.992 |
|  | summer female - winter male | 0.41 | 0.08 | 77.00 | 5.43 | 0.001 |
|  | winter female - summer male | 0.08 | 0.07 | 77.00 | 1.11 | 0.682 |
|  | winter female - winter male | 0.51 | 0.07 | 77.00 | 6.98 | 0.001 |
|  | summer male - winter male | 0.43 | 0.07 | 77.00 | 5.84 | 0.001 |
| Simpson | summer female - winter female | 0.00 | 0.00 | 77.00 | 1.09 | 0.697 |
|  | summer female - summer male | -0.00 | 0.00 | 77.00 | -0.09 | 0.999 |
|  | summer female - winter male | -0.01 | 0.00 | 77.00 | -2.59 | 0.054 |
|  | winter female - summer male | -0.00 | 0.00 | 77.00 | -1.21 | 0.619 |
|  | winter female - winter male | -0.01 | 0.00 | 77.00 | -3.77 | 0.001 |
|  | summer male - winter male | -0.01 | 0.00 | 77.00 | -2.56 | 0.058 |
